# 85 The Severity of Burns and Associated Psychiatric Risk Factors in Self-Immolation

**DOI:** 10.1093/jbcr/irad045.059

**Published:** 2023-05-15

**Authors:** Srinagesh Mannekote Thippaiah, Gilbert Ramos, Karen Richey, Kevin Foster, Ibraheim Ayub, Youssef Challita

**Affiliations:** Arizona Burn Center Valleywise Health, Phoenix, Arizona; Arizona Burn Center Valleywise Health, Phoenix, Arizona; Arizona Burn Center Valleywise Health, Phoenix, Arizona; AZ Burn Center at Valleywise Health, Cave Creek, Arizona; Arizona Burn Center Valleywise Health, SCOTTSDALE, Arizona; Valleywise Health Medical Center, Phoenix, Arizona; Arizona Burn Center Valleywise Health, Phoenix, Arizona; Arizona Burn Center Valleywise Health, Phoenix, Arizona; Arizona Burn Center Valleywise Health, Phoenix, Arizona; AZ Burn Center at Valleywise Health, Cave Creek, Arizona; Arizona Burn Center Valleywise Health, SCOTTSDALE, Arizona; Valleywise Health Medical Center, Phoenix, Arizona; Arizona Burn Center Valleywise Health, Phoenix, Arizona; Arizona Burn Center Valleywise Health, Phoenix, Arizona; Arizona Burn Center Valleywise Health, Phoenix, Arizona; AZ Burn Center at Valleywise Health, Cave Creek, Arizona; Arizona Burn Center Valleywise Health, SCOTTSDALE, Arizona; Valleywise Health Medical Center, Phoenix, Arizona; Arizona Burn Center Valleywise Health, Phoenix, Arizona; Arizona Burn Center Valleywise Health, Phoenix, Arizona; Arizona Burn Center Valleywise Health, Phoenix, Arizona; AZ Burn Center at Valleywise Health, Cave Creek, Arizona; Arizona Burn Center Valleywise Health, SCOTTSDALE, Arizona; Valleywise Health Medical Center, Phoenix, Arizona; Arizona Burn Center Valleywise Health, Phoenix, Arizona; Arizona Burn Center Valleywise Health, Phoenix, Arizona; Arizona Burn Center Valleywise Health, Phoenix, Arizona; AZ Burn Center at Valleywise Health, Cave Creek, Arizona; Arizona Burn Center Valleywise Health, SCOTTSDALE, Arizona; Valleywise Health Medical Center, Phoenix, Arizona; Arizona Burn Center Valleywise Health, Phoenix, Arizona; Arizona Burn Center Valleywise Health, Phoenix, Arizona; Arizona Burn Center Valleywise Health, Phoenix, Arizona; AZ Burn Center at Valleywise Health, Cave Creek, Arizona; Arizona Burn Center Valleywise Health, SCOTTSDALE, Arizona; Valleywise Health Medical Center, Phoenix, Arizona

## Abstract

**Introduction:**

Self-Immolation is the act of setting oneself on fire to inflict death or self-injury. It is most commonly seen in developing nations and is associated with high mortality. In developed countries, self-immolation is uncommon and occurs mostly in middle-aged males with a history of mental illness, substance abuse, and unemployment. In the United States, it is an extreme act of suicide or self-injurious behavior with a mortality rate of 11.4% and exhibits a significant burden on healthcare systems. In this study authors aimed to characterize and explore psychiatric risk factors for self-immolation; broader understanding helps to optimize patient care and reduce the future recurrence of self-immolation act.

**Methods:**

This was a retrospective review of all inpatient admissions from July 2015 to August 2022 with a history of self-immolation were included in the study. Data collection included demographics, total body surface area burned (TBSA), Suicide attempt or Self injurious behavior, urine drug screen, length of stay and psychiatric history. Twelve patients without incomplete data were excluded. Patients were divided into mild (< 20%), moderate (20-40%), and severe ( >40%) burns based on percent of TBSA. Data analysis was conducted using Chi-Squared, and Fisher's Exact tests in SPSS v28.

**Results:**

We identified 103 patients who attempted self-immolation. Mean TBSA was 27 ± 33 with 66 (64%) experiencing mild, 11 (10%) with moderate, and 27 (26%) with severe burns. Mortality in this population was 21%. The majority were male (70.9%), Caucasian (63.1%), and single (79.6%) with a mean age of 38 ± 14 years old. Urine drug screen was positive in 44% upon admission, and 63% reported chronic drug use. Methamphetamine (37%), and alcohol (30%) were in the highest prevalence upon admission. Additionally, 83% of patients had underlying psychiatric illnesses including major depressive disorder (36%) and Schizophrenia spectrum disorders (27%). The presence of suicidal intent (n=68) was a predicted risk factor for severe burns (p < 0.001).

**Conclusions:**

Our study found that patients undergoing self-immolation were more likely to have suicidal intent and that suicidal intent strongly predicted burn severity. Although no specific psychiatric disorder predicted burn severity, the overwhelming majority of self-immolation patients suffered from underlying psychiatric illnesses.

**Applicability of Research to Practice:**

There is limited data on the triggers and risk factors for self-immolation. Due to the large prevalence of drug use and underlying psychiatric disorders, providers should obtain an early psychiatric assessment for self-immolation patients to minimize recurrence and optimize inpatient care. Similarly, home safety education for patients with suicidal intent and their families may help reduce the frequency and severity of self-immolation in the outpatient setting.

